# Complex chloroplast RNA metabolism: just debugging the genetic programme?

**DOI:** 10.1186/1741-7007-6-36

**Published:** 2008-08-28

**Authors:** Uwe G Maier, Andrew Bozarth, Helena T Funk, Stefan Zauner, Stefan A Rensing, Christian Schmitz-Linneweber, Thomas Börner, Michael Tillich

**Affiliations:** 1 Philipps University Marburg, Cell Biology, Karl-von-Frisch Str., D-35032, Marbur, Germany; 2 University of Freiburg, Faculty of Biology, Schaenzlestr. 1, D-79104, Freiburg, Germany; 3 Humboldt University Berlin, Institute of Biology, Chausseestr. 117, D-10115, Berlin, Germany

## Abstract

**Background:**

The gene expression system of chloroplasts is far more complex than that of their cyanobacterial progenitor. This gain in complexity affects in particular RNA metabolism, specifically the transcription and maturation of RNA. Mature chloroplast RNA is generated by a plethora of nuclear-encoded proteins acquired or recruited during plant evolution, comprising additional RNA polymerases and sigma factors, and sequence-specific RNA maturation factors promoting RNA splicing, editing, end formation and translatability. Despite years of intensive research, we still lack a comprehensive explanation for this complexity.

**Results:**

We inspected the available literature and genome databases for information on components of RNA metabolism in land plant chloroplasts. In particular, new inventions of chloroplast-specific mechanisms and the expansion of some gene/protein families detected in land plants lead us to suggest that the primary function of the additional nuclear-encoded components found in chloroplasts is the transgenomic suppression of point mutations, fixation of which occurred due to an enhanced genetic drift exhibited by chloroplast genomes. We further speculate that a fast evolution of transgenomic suppressors occurred after the water-to-land transition of plants.

**Conclusion:**

Our inspections indicate that several chloroplast-specific mechanisms evolved in land plants to remedy point mutations that occurred after the water-to-land transition. Thus, the complexity of chloroplast gene expression evolved to guarantee the functionality of chloroplast genetic information and may not, with some exceptions, be involved in regulatory functions.

## Background

**Figure 1:**
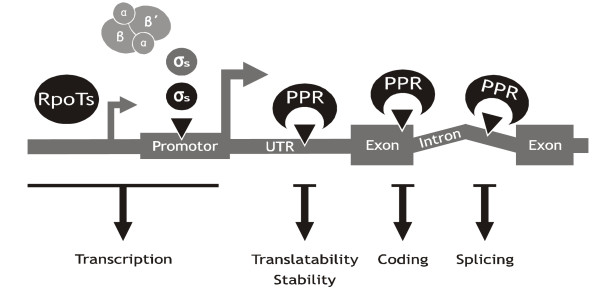
**Components of the ancient basal (cyanobacterial-like, grey) and modern extended (grey and black) gene expression system of chloroplasts**. We propose that the evolution of RpoTs in plants, the expansion of the gene families coding for chloroplast sigma factors and PPR proteins help to neutralize mutational lesions in the chloroplast genomes (black triangles). RpoTs and additional sigma factors with lower or altered promoter specificity compensate for degenerated promoters. The highly specific members of the versatile PPR family promote or are required for proper translation, coding and splicing of chloroplast mRNAs.

As the site of oxygenic photosynthesis, chloroplasts are the most in-depth studied plant organelles. The recognition that they carry their own DNA [1] led, in the 1970s, to a race to decipher their genetic content, which eventually resulted in more than 100 [2] sequenced chloroplast genomes to date. These sequences set to rest any remaining doubts that chloroplasts are ancient endosymbionts and are derived from cyanobacterial-like ancestors. Expression analysis of the chloroplast genetic information had been studied right from the dawn of chloroplast molecular biology and several surprising findings emerged. The most puzzling of these was that the chloroplast gene expression system is far more complex than that of its cyanobacterial progenitors (Figure 1). This gain in complexity is due to changes in RNA metabolism, specifically to novelties in the transcription and maturation of RNA. A set of newly acquired or recruited nuclear-encoded proteins comprising RNA polymerases, sigma factors and mono- or merospecific RNA maturation factors promotes transcription [3-8], RNA splicing [9-11], RNA editing [12-14], RNA end formation [15-17] or translation [18,19]. Identification and characterization of these factors stimulated research, but a unifying explanation for this Byzantine gene expression system in chloroplasts has not yet been found. Here, we propose a hypothesis on the origin of the complexity of chloroplast gene expression, encompassing recent data on factors involved in chloroplast transcription, RNA editing and RNA processing.

## Results and discussion

### Amazing complexity: chloroplast RNA transcription, RNA processing and the corresponding machinery

The chloroplast genome contains functional *rpo *genes encoding subunits of a homolog of the eubacterial RNA polymerase, termed plastid-encoded RNA polymerase (PEP). Surprisingly, in contrast to its eubacterial ancestors, this RNA polymerase does not transcribe the chloroplast genome in higher plants alone [20-22]. A second, chloroplast-localized, but nuclear-encoded transcription activity, the nuclear-encoded plastid RNA polymerase (NEP), has been identified (Figure 1), which has promoter requirements very different from the canonical -10/-35 PEP promoters. Sequence alignments revealed that most NEP promoters contain a simple core sequence (YRTA), similar to plant mitochondrial promoters. NEP activity consists of one (Liliopsida) or two (eudicotyledonous plant species) phage-type RNA polymerases [23,24]. These three RNA polymerases produce a dazzling array of transcripts using a multitude of different promoters. As a consequence, almost all chloroplast genes are transcribed from several independent promoters [25].

Similarly to transcription, RNA processing also dramatically increases the variety of transcripts per gene. This is reflected in the complex transcript patterns encountered when performing chloroplast RNA gel blot hybridization experiments. More than 10 transcripts per gene, differing in size and in their coverage of adjacent cistrons, are regularly detected. Additionally, alternative RNA cleavage can produce mature RNAs differing in their translational efficiency [26]. All of this is not known from transcripts in cyanobacteria. Mostly, splicing and cleavage events in chloroplasts cause the breakdown of their long precursor RNAs into monocistronic constituents. For example, there are five precursor RNA species for *ndhA *and as many as 15 for *petD *[27,28]. Both genes carry an intron and are part of a primary precursor encompassing seven and four genes, respectively. Another curiosity adding to the overall complexity of chloroplast RNA processing is RNA editing. In seed plants, about 35 C-to-U editing sites are found per chloroplast genome [summarized in [29,30]], whereas chloroplast genomes of bryophytes like those of the hornwort *Anthoceros *can harbour several hundred sites [31]. In cyanobacteria, RNA editing is not observed at all. Furthermore, splicing has a less prominent role in RNA maturation in these prokaryotic relatives of the chloroplast ancestor [32,33]. Although endonucleolytic cleavage events are important in bacterial RNA degradation, they have not been demonstrated to be a common prerequisite for translation [34]. Thus, complexity in RNA processing arose after endosymbiosis. Given that hundreds of processing events occur, a legitimate question is: who does the job? Certainly, the vast majority of factors involved are nuclear-encoded. One family that came into focus recently as an important player in plant organellar RNA metabolism is the pentatricopeptide repeat (PPR) protein family. Many of its members (about 100 in rice and *Arabidopsis*) are targeted into chloroplasts [35]. Those that have been analysed genetically are required for specific steps in chloroplast gene expression. PPRs recognize their targets with high specificity [10,19] and are direct ligands of RNAs [13,14,36,37]. They are currently viewed as the adaptors that are necessary for the myriad sequence-specific processing events occurring in chloroplast transcripts [35,38]. Besides PPRs, a variety of additional factors of different specificity have been described that execute processing steps left over by PPR proteins [39-41]. In conclusion, chloroplasts are engaged in a tremendous expenditure of matter and energy to generate multiple RNA species. No clear function is known either for the array of transcripts produced or for the complex transcription and RNA processing itself.

### Positive selection for complexity or haphazard accumulation of processing events?

Undoubtedly, many steps in chloroplast RNA metabolism are essential for gene expression; e.g. all splicing events and most editing events. But does 'essential' mean that these processing steps are under positive selection? Or in other words: is there evidence that these processing steps fulfil some sort of regulatory function; i.e. are they rate-limiting for chloroplast gene expression? The currently available data are not adequate to decide in favour of or against this question. This is mostly due to the fact that it is non-trivial to demonstrate unequivocally that a specific transcription initiation, splicing, or editing event is limiting for the amount of the final gene product. While it is an appealing idea that the multitude of transcription and processing steps mediated by nuclear encoded factors represent starting points for the regulation of organelle gene expression and its nuclear control [42], there is only a single study directly supporting this argument, carried out recently in *Chlamydomonas reinhardtii*. Here, the abundance of the chloroplast PPR protein MCA1 has been demonstrated to limit the amount of the *petA *mRNA as well as the amount of the encoded protein, cytochrome f [43]. In plants, only marginal evidence for the regulation of gene expression by factors involved in controlling RNA metabolism is available [e. g. [44]]. For example, organellar RNA polymerases have been shown to be expressed in a tissue-specific manner and seem to be modified dependent on light conditions [45,46]. A variety of studies show that transcription and post-transcriptional events can be modulated by external factors like temperature and light-conditions [47-49]. None of these studies, however, yielded conclusive evidence as to whether gene expression is truly rate-limiting. Several authors have suggested that there is a dominance of translational and post-translational regulation of gene expression in chloroplasts [50,51]. In contrast to pre-translational regulation, there is ample evidence for the regulation of translation or subsequent processes, both in *C. reinhardtii *and seed plants [52-55]. Taken together, currently there is little evidence that the complexity of organellar RNA metabolism is a result of an increased demand for regulating steps in gene expression, although it cannot be ruled out that we just have not looked hard enough.

### An alternative explanation: complexity as a result of transgenomic suppression?

Although positive selection of a complex RNA metabolism of chloroplasts remains a potent possibility for explaining its having come about, we think that there is at least one powerful alternative explanation. In short, this hypothesis puts forth the proposition that complexity in organellar gene expression can be a selectively neutral process, fostered by different mutation rates and a different strength of genetic drift between the organellar and nuclear genomes of plants.

Chloroplasts harbour multiple copies of their genome and are inherited clonally, thus recombination between identical copies has an insignificant role in chloroplast evolution [56]. These are the hallmarks of a degenerative process occurring in genomes of endoparasites, termed Muller's ratchet [57,58]. Genomes of organisms like the proteobacterium *Buchnera*, an endosymbiont of aphids, accumulate mildly deleterious mutations [59]. As endosymbionts, chloroplasts should likewise be prone to the same type of genomic degeneration. Of course, countermeasures may evolve in endosymbionts to lighten the mutational burden imposed by Muller's ratchet, like modifications of the DNA repair system [60]. While the welfare of *Buchnera *depends on such intrinsic relief systems, chloroplasts can draw on a tremendous external resource for treating genomic problems: they can tap the coding potential of the nuclear genome. The plant nuclear genome is much more dynamic and evolves more rapidly than the chloroplast genome, among other reasons because of the benefits of sexual recombination and a higher mutation rate [61-63]. However, the situation might differ in bryophytes and ferns, in which the dominant generation is haploid. Nevertheless, recent results indicate that as in seed plants, the organellar genomes of ferns and bryophytes exhibit lower mutation rates than their nuclear counterparts [64].

The nuclear genome provides a wealth of genetic information that can be directed into the organelle with relative ease [65,66]. This way, the more expeditious evolution of nuclear loci could alleviate slow deterioration of chloroplast genetic information, and even mutations with a strong negative impact on organelle development and function might be remedied. We view this compensatory process as being suppressor mutations that do not reverse the original point mutation but find an alternative way to correctly decode the chloroplast genetic information. Thus, chloroplast mutations and nuclear suppressors form co-evolving couples, reciprocally stabilizing each other. This phenomenon is not a mere academic speculation, but has been witnessed in our time by plant breeders. Cytoplasmic male sterility (CMS) is a defect occurring in plant mitochondria that leads to loss of pollen development and thus, male sterility. CMS plant lines have been isolated in many different agronomically important crops. In most cases, illegitimate recombination events in the mitochondrial genome resulted in novel open reading frames (ORFs), the expression of which is detrimental to mitochondrial function. Plant breeders take advantage of CMS lines for their large-scale breeding programmes, because carrier plants do not require emasculation for out-crossing. For many CMS populations, nuclear loci that restore fertility are described. These restorers are specific to their cognate mitochondrial defect. Eight restorer genes have been cloned [summarized in [67]]. All but one of them encode PPR proteins targeted to mitochondria. For rice RF1A it was shown that it leads to endonucleolytic cleavage of the aberrant CMS RNA [68]. In these cases, a nuclear factor alleviates an organellar problem arising due to a genomic mutation.

We propose that transgenomic suppression silences deleterious chloroplast point mutations in all coding sequences as well as in sequence elements required for gene expression. In the following section, we discuss which nuclear factors are models for suppression of chloroplast mutations. We propose that PPR proteins are involved in neutralizing point mutations in coding regions, introns and UTRs, while different RNA polymerases and their co-factors help overcome problems caused by point mutations in promoter regions.

### PPRs counteract point mutations in coding regions, introns and UTRs

As outlined above, some PPRs are restorers of fertility that counteract detrimental mutations in mitochondria. In chloroplasts, PPR proteins fulfil a variety of functions in RNA metabolism. PPR proteins are involved in RNA editing [12-14], RNA splicing [10] and RNA cleavage [36,69]. Most plant PPR proteins analysed to date are essential proteins. However, no evidence for their being involved in regulating gene expression has been presented thus far, underscored by the fact that most PPR proteins are constitutively expressed [35]. We hypothesise that PPRs are a nuclear remedy of mutations occurring in the chloroplast genome. This is best exemplified by their role in CMS (see above) and plastid RNA editing.

Several lines of evidence suggest that chloroplast RNA editing is one mechanism for compensating damage from point mutations in the chloroplast chromosome. RNA editing has been demonstrated to be essential: the corresponding gene product malfunctions if Cs are not turned into Us on the RNA level. Contrasting its importance for gene function, RNA editing *per se *is not under positive selection, as indicated by the extraordinary speed with which editing sites evolve [70], and by the fact that experimental removal of a site did not lead to any discernible phenotype [71]. Furthermore, RNA editing sites are most stable in a base context that exhibits low mutation rates. In particular, editing sites seem to be stuck in the T_A context [30]. Cs squeezed between a 5' T and a 3' A have the lowest mutation rates when compared with any other possible immediate C-neighbourhood [72]. It is precisely this context that is most frequently encountered around editing sites. Moreover, editing sites accumulate predominantly in genomic regions of the chloroplast chromosome that are slowly evolving, foremost the inverted repeat [30,73]. Yet, if RNA editing was under positive selection, a correlation of editing sites with local mutation rates would not be expected. Together, these data suggest that RNA editing sites arose as T-to-C mutations that are not easily removed by mutational reversion. In conclusion, RNA editing is not present in chloroplast genomes for the benefit of regulating gene expression, but rather for getting rid of mutations at the RNA level [30].

Recently, it has been shown that, at least in well-investigated cases, the nuclear encoded factors responsible for recognizing editing sites are PPR proteins [reviewed in [67]]. Thus, RNA editing sites and their cognate editing PPR proteins exactly mirror the situation of CMS mutations and nuclear restorer PPR genes. Again, a nuclear-encoded PPR protein seems to be a remedy for an organellar genomic problem.

As mentioned above, PPR target sites are not restricted to coding regions; neither are point mutations due to organellar DNA degeneration. Organellar introns for instance are highly divergent from their bacterial ancestors [74]. The dominant class of introns in chloroplasts is the so-called group II introns, which fold into a characteristic secondary structure. In bacteria, group II introns are self-splicing *in vitro *and are by definition ribozymes. Chloroplast group II introns, however, have lost their ability to excise without external help. This might also point to the detrimental effects of Muller's ratchet being active in chloroplasts. For splicing, nuclear encoded factors are required [reviewed in [75]]. Interestingly, several of these factors are PPR proteins [9-11]. It seems superfluous to invent novel splicing factors for each intron. We deem it more likely that similar to the situation with RNA editing, nuclear factors, foremost PPR proteins, help to counterbalance organellar mutations. For example, secondary structure elements in group II introns disrupted by point mutations could be stabilized by the interaction with PPR proteins.

Besides coding regions and introns, Muller's ratchet should also lead to the weakening or even destruction of expression signals in the 5' and 3' UTR of mRNAs. Sequence elements in the UTR regions are required for RNA stability, but (particularly in the 5' UTR) also for translation initiation. Indeed, the canonical Shine Dalgarno sequence in front of cyanobacterial genes is rarely found in the typical position in front of chloroplast open reading frames, if it is found at all [reviewed in [76,77]]. Chloroplasts have developed alternative schemes to load ribosomes onto their mRNAs that interact with specific nuclear RNA binding proteins [78]. Not surprisingly, PPR proteins also play a part in this task [18,19].

### Multiple RNA polymerases with relaxed promoter specificities counteract point mutations in promoter regions

Many point mutations can be cured on the RNA level by nuclear factors, i.e. highly specific RNA binding proteins. In contrast, processes depending on DNA as a template will require other means to compensate for deleterious point mutations. These DNA-bound processes are chiefly replication and transcription. Only some features of the replication machinery in chloroplasts are known (e.g. bacterial-type DNA polymerases [79]) but, clearly, bacterial origins of replication are no longer found on chloroplast chromosomes [80], putatively eliminated by Muller's ratchet. Much more is known about transcription initiation and the responsible factors.

In cyanobacteria, promoters of the -10/-35 type are used to drive the transcription of all genes. More than a dozen sigma factors are found per cyanobacterial genome that fall into different functional classes with different consensus sequences in the -10 and -35 boxes [e. g. NC_000911 [81,82]]. In plants, sigma factors are no longer encoded by the chloroplast genome but their genes have been transferred into the nucleus. The number of sigma factors found in the *Arabidopsis thaliana*, grapevine (*Vitis vinifera*), rice (*Oryza sativa*) and moss *Physcomitrella patens *genome is six; nine were found in poplar (*Populus trichocarpa*), but only one is present in the sequenced green algal genomes of *C. reinhardtii*, *Ostreococcus tauri *and *Ostreococcus lucimarinus *(Table 1). This represents a remarkable multiplicity in sigma factors in land plants compared with green algae and a dramatic increase in their number per gene relative to cyanobacteria, considering the small number of chloroplast genes (Table 1). While sigma factors are notorious suspects for gene regulation and indeed have at least in part taken over regulatory functions [reviewed in [44,83]], the increase in nuclear-encoded sigma factors for chloroplast genes' promoters could originally have been a reaction to promoter degeneration, i.e. diversification. In fact, canonical -10/-35 promoters are rare in chloroplasts. Both a pronounced deviation from the consensus and a spatial shift from the start codon are common. Thus, multiple sigma factors may be needed to recognise deviant promoters in chloroplasts. Remarkably, at least two sigma factors, SIG3 and SIG4, are highly specific and seem to serve only the transcription of a single gene, *psb*N and *ndh*F, respectively [44,84,85]. This high specificity resembles the situation with PPRs and their specific targets and – in the absence of data indicating regulation for these two sigma factors – might be a sign that at least these two sigma factors are nuclear solutions to cope with a degenerate chloroplast promoter.

**Table 1:** Compilation of the number of nuclear encoded, chloroplast directed transgenomic suppressor functions (PPR proteins, sigma factors and phage type RNA polymerases) and of plastid genes encoded among plants and unicellular algae.

	***A. thaliana***	***P. trichocarpa***	***V. vinifera***	***O. sativa***	***P. patens***	***C. reinhardtii***	***O. tauri***	***O. lucimarinus***	***T. pseudonana***	***P. tricornutum***	***C. merolae***
**PPR proteins**	482	641	610	491	110	13	19	22	46	50	8
**Sigma 70 factors**	6	9	6	6	6	1	1	1	4	5	4
**Plastid genes**	85	99	84	64	85	69	43	n.a.	141	132	207
PPR/plastid gene*	0.90	1.04	1.16	1.23	0.21	0.03	0.07	n.a.	0.05	0.06	0.006
**Phage-type RNA polymerases**	3	>3	3	2	3	1	1	1	1	1	1

*The PPR protein-plastid gene relations were determined under the assumption that in all taxa 16% of the PPR proteins are plastid proteins (as shown for *A. thaliana *in [35]).

n.a. = not applicable.

A second transcription system in chloroplasts appears even more suitable to avert problems arising from mutated promoters: the nuclear encoded phage-type RNA polymerases (NEP). Angiosperms possess one or two phage-type RNA polymerases in their chloroplasts, which are needed for transcription in addition to PEP. NEP and PEP recognise different promoters and PEP is discussed as playing a major role in green tissues [44,83]. A thorough analysis of NEP promoter function (*rpoB *promoter of tobacco) revealed a CRT-motif (CAT or CGT) at position -8 to -6, which was proven to be critical for transcription [86]. Such a simplistic core promoter of only three nucleotides is expected to be found upstream, proximal to each transcripional unit. Moreover, phage-type NEP enzymes are likely able to recognise promoter sequences without the help of auxiliary factors. The T7 RNA polymerase is known to operate as a single subunit enzyme, i.e. one and the same polypeptide performs promoter recognition and all phases of transcription [87]. At least *in vitro*, the mitochondrial RNA polymerase of baker's yeast exhibits similar properties, although efficient transcription requires two additional protein factors *in organello *[88]. Similarly, the *A. thaliana *phage-type enzymes, including the mitochondrial and a chloroplast-targeted RNA polymerase, were found to act as single-polypeptide transcriptases and to recognise several mitochondrial and chloroplast promoters *in vitro *[89]. These observations suggest that the targeting of a phage-type RNA polymerase to chloroplasts alone, i.e. without additional transcription factors, might have been sufficient to support transcription from promoters with simple structures. Thus, NEP transcription initiation may avert detrimental effects of point mutations by decreasing specificity. This is a fundamentally different strategy from the one that PPR proteins and sigma factors follow in order to counteract point mutations, as apparently they function with high specificity. In summary, the complexity of the transcription apparatus in chloroplasts could have evolved to compensate for degenerating chloroplast promoters.

### Emergence of land plants and transgenomic suppressors coincide

Chloroplast DNA mutation and degeneration should be an ongoing process and the example of restorer genes and CMS highlights that suppression of organellar mutations still occurs in today's plants. However, when examining the different transgenomic suppressors enumerated above under a more phylogenetic perspective, an intriguing bias towards land plants emerges (see Table 1). First, RNA editing is an invention of land plants, absent from algae [90]. Second, although present in all eukaryotic genomes including algae (albeit in low numbers in those: 19 to 26 members, see Table 1), PPR proteins are exceptionally abundant in land plants. More than 450 members have been identified in *A. thaliana *and rice, more than 600 in poplar and grapevine, and approximately 100 in the moss *P. patens *(Table 1) [9]. Furthermore, whereas the genomes of the green algae *C. reinhardtii*, *O. tauri *and *O. lucimarinus *encode only one sigma factor, the seed plant and *P. patens *genomes encode at least six [44]. Finally, seed plants and mosses have evolved plastidal NEPs by duplicating the gene encoding the mitochondrial phage-type enzyme [4,91], while genomes from green algae and the red alga *Cyanidioschyzon merolae *only encode one phage-type RNA polymerase [44], which is predicted to be targeted to the mitochondrion. Although some nucleus-encoded factors involved in RNA maturation are known from *Chlamydomonas *[92], an apparently shared feature of land plants is an increase in proteins that could function in sustaining the functionality of mutationally altered organellar genomes. How can this trend be explained?

Today it is generally accepted that land plants share a common ancestor with the green algal lineage [93-95]. Because land plants are monophyletic [96,97], one has to assume that a single green algal-like progenitor successfully went ashore, subsequently adapted to a dramatically different environment, and eventually evolved into the earliest land plant.

Changing from an aquatic to a terrestrial life style must have been a colossal challenge in several regards, yet the colonisation of land by plants was nonetheless very successful. The evolutionary radiation into mosses, liverworts, hornworts, club mosses, ferns and seed plants was accompanied by major developmental and morphological innovations, enabling the adaptation of plants to the new environment [97-99]. Coevally to the need for the development of new morphotypes, early land plants required adaptations to cope with the exposure to increased solar and stellar radiations, which endangered their genetic information. Conceivably, this increased the need for countermeasures against chloroplast mutations and eventually led to the expansion of the PPR family, the increase in sigma factors per gene, and the redirection of a phage-type RNA polymerase to chloroplasts. If this hypothesis is true, we should see clear differences in the number of PPR proteins, RNA editing and RNA polymerases not only between green algae and land plants, but also between more closely related sister groups of land plants, for example the charophytes. Further genomic information is needed to resolve these questions.

As shown for RNA-editing, species-specific gains and losses of mutations are common as well, which necessitate restoration by nucleus-encoded factors. In the case that losses of mutation sites are predominant, suppressor activity for that mutation can be lost coevally. A very pronounced situation for the loss of a plant-specific mechanism is present in the liverwort *Marchantia polymorpha*, which secondarily lost organellar RNA editing (as shown recently by the identification of RNA editing within the mitochondrion of an ancestral liverwort [100]). Thus, in the case of a high mutation rate enabling back-mutations or the evolution of additional transgenomic suppressors, other land plant-specific mechanisms such as a second, nucleus-encoded chloroplast RNA polymerase should ultimately become superfluous and be lost.

## Conclusion

Here, we put forward a hypothesis to account for the enigmatic complexity of chloroplast gene expression. We propose that nuclear factors evolved to counteract chloroplast mutations that occurred after the water-to-land transition and persist due to the mode of chloroplast genome evolution. These nuclear factors act either on DNA directly or suppress point mutations on the RNA level. Specifically, we suggest that PPR proteins were recruited to counteract point mutations in coding regions, introns and UTRs. In the same way, sigma factors may help to recognise degenerated promoter motifs. Finally, low-specificity phage-type RNA polymerases support transcription despite the loss of canonical PEP-promoters. Intriguingly, all means to suppress chloroplast mutations instanced here seem to have evolved in early land plants. Hypothetically, this could mean that chloroplast genomic decay and parallel counteraction by nuclear-encoded components were accelerated in the common ancestor of all land plants.

The hypothesis put forward here draws on two established concepts: the degeneration of genomes of endosymbiotic organisms and the suppression of organellar defects by nuclear factors (the CMS example). The hypothesis circumvents the need to invoke selective pressures to account for the myriads of processing events in the chloroplast transcriptome (but does not exclude them in individual cases). It is therefore more parsimonious than the assumption that complexity in chloroplast gene expression serves a regulatory function. Finally, our hypothesis may draw a picture of the first land plant, its molecular integration into the new environment and the need to suppress radiation-engendered mutations.

## Methods

Available genome datasets were screened with the following PFAM () HMMs with a cutoff of *E *≤ 0.0001: Sigma70_r2/3/4 (PF 04542, 04539, 04545); PPR repeat (PF01535). Only those genes containing more than one PPR repeat were taken into account. Numbers of protein-coding plastid genes are derived from [2]. Number of *RpoT *genes encoding phage-type RNA polymerases in *O. tauri, O. lucimarinus, T. pseudonana *and *P. tricornutum *are derived from BLAST hits using the *A. thaliana *RpoTm, RpoTmp and RpoTp sequences as queries, filtered for > = 30% identity and 300 aa alignment length. *RpoT *gene numbers in other organisms were taken from the literature [cf. [44]] or communicated by Andreas Weihe and Uwe Richter (Humboldt University, Berlin).

## Authors' contributions

UGM conceived the study. SAR searched the available data for transgenomic suppressors. All authors discussed the results, wrote and approved the final manuscript.
